# The Early Symptoms of Insular Sclerosis

**Published:** 1905-08-12

**Authors:** 


### THE EARLY SYMPTOMS OF INSULAR SCLEROSIS.

In an interesting paper on this subject Dr. E. Hobhouse [1] insists that insular sclerosis is the most common form of chronic nervous disease likely to be met with in general medical practice. In addition to the cases which are recognised it is probable that not a few instances in which the diagnosis is marked, neurasthenia, hysteria, or neurosis, are really early cases of insular sclerosis. A feature of the disease of great interest is the diversity of the symptoms which it presents. Hence early cases may come under the attention, not of the physician, but of the surgeon or gynæcologist or ophthalmic surgeon. This variety in the symptoms, too, means that the diagnosis has often to be made without the presence of some of the most typical and characteristic clinical evidence of the disease. For the most part insular sclerosis commences with weakness and trembling in the limbs, but not infrequently the early symptoms are associated with the sense of sight. Thus in one of Dr. Hobhouse's cases the first evidence of the disease was ophthalmoplegia externa, which, after lasting four months, suddenly disappeared; in a second, there was sudden but temporary loss of sight in one eye; in a third, the same experience preceded weakness of the legs by a period of six years; and in two others double vision was an early event. Further illustrations of the various forms in which the disease first shows itself are found in examples of tinnitus, aphonia, disturbance of the functions of the bladder, and hysterical contracture. A point of great importance is to remember that in many instances symptoms readily regarded as hysterical are really early facts in the evolution of insular sclerosis. Dr. Hobhouse further suggests that menorrhagia may be one of the first symptoms of the disease. He has noticed this in several instances and thinks it may be due to involvement of the lumbar centres. In reference to hysterical manifestations the transitory nature of the symptoms is often very marked. Not only are patients sometimes very emotional, but such definite evidences as paralysis in one or more limbs may be of purely temporary duration, and may thus appear to be of functional origin. The same thing is true in reference to sight. Sudden blindness may come on one day and disappear after a very short duration. Such events, especially if recurrent, ought always to suggest the possibility of insular sclerosis. The lapse of time without organic symptoms may prove the case to be one of hysteria, but not infrequently it brings conclusive proof of organic disease. In one of Dr. Hobhouse's cases, the patient, a woman, aged 39, had for several years been regarded as a typical example of hysteria. Amongst other symptoms she had been treated for hysterical contracture and for an ulcer on the abdomen, the latter almost certainly self-inflicted. Yet later there appeared definite evidences of insular sclerosis. In another case there had been anæsthesia and vomiting, both of which were treated as functional events. There had also been a sudden amblyopia of 24 hours' duration. After a time, however, tremors in the limbs, loss of sphincter control, and an exaggeration of the tendon phenomena, showed the case to be an undoubted example of insular sclerosis. These and other cases quoted in Dr. Hobhouse's article exactly accord with the propositions which Dr. Buzzard has done so much to illustrate and enforce.

[1] Lancet, Feb. 18, 1905.

